# Noninvasive Diagnosis of Hepatic Fibrosis in Hemodialysis Patients with Hepatitis C Virus Infection

**DOI:** 10.3390/diagnostics12102282

**Published:** 2022-09-21

**Authors:** Chen-Hua Liu, Jia-Horng Kao

**Affiliations:** 1 Department of Internal Medicine, National Taiwan University Hospital, Taipei 100225, Taiwan; 2 Hepatitis Research Center, National Taiwan University Hospital, Taipei 100225, Taiwan; 3 Department of Internal Medicine, National Taiwan University Hospital, Yun-Lin Branch, Douliou 640203, Taiwan; 4 Graduate Institute of Clinical Medicine, National Taiwan University College of Medicine, Taipei 100233, Taiwan; 5 Department of Medical Research, National Taiwan University Hospital, Taipei 100225, Taiwan

**Keywords:** hepatitis C virus, hemodialysis, noninvasive diagnosis, hepatic fibrosis

## Abstract

Hepatitis C virus (HCV) is a major health problem in hemodialysis patients, which leads to significant morbidity and mortality through progressive hepatic fibrosis or cirrhosis. Percutaneous liver biopsy is the gold standard to stage hepatic fibrosis. However, it is an invasive procedure with postbiopsy complications. Because uremia may significantly increase the risk of fatal and nonfatal bleeding events, the use of noninvasive means to assess the severity of hepatic fibrosis is particularly appealing to hemodialysis patients. To date, researchers have evaluated the performance of various biochemical, serological, and radiological indices for hepatic fibrosis in hemodialysis patients with HCV infection. In this review, we will summarize the progress of noninvasive indices for assessing hepatic fibrosis and propose a pragmatic recommendation to diagnose the stage of hepatic fibrosis with a noninvasive index, in hemodialysis patients with HCV infection.

## 1. Introduction

Hepatitis C virus (HCV) infection, which may result in fibrosis, cirrhosis, hepatic decompensation, and hepatocellular carcinoma (HCC), is a leading cause of chronic liver disease in patients receiving hemodialysis [1,2,3,4]. In addition to a solid link to liver-related morbidities, HCV infection is associated with a high risk of cardiovascular and infectious-related hospitalization and mortality in hemodialysis patients [5]. In contrast, the health-related outcomes are significantly improved once HCV is eradicated with effective antiviral treatment [6,7,8,9,10]. Because nearly all patients can successfully clear HCV infection with a short course of potent and safe direct-acting antivirals (DAAs), they are particularly relevant to practitioners and hemodialysis patients in moving toward HCV microelimination by 2030 [11,12,13,14,15,16,17,18].

Although the introduction of DAAs has tremendously advanced HCV care, accurate staging of hepatic fibrosis remains essential for therapeutic and prognostic purposes. The presence of cirrhosis may affect the treatment duration, the need for ribavirin (RBV) coadministration, and the sustained virologic response (SVR) rates in certain groups of patients [19,20,21]. Furthermore, information about the severity of hepatic fibrosis can efficiently help clinicians determine the surveillance strategies for portal hypertension and HCC before and after viral cure [22,23,24,25]. Currently, percutaneous liver biopsy is the gold standard to stage hepatic fibrosis. However, it is an invasive procedure with poor patient acceptance. Because platelet dysfunction significantly affects hemostasis in kidney failures, hemodialysis patients with HCV infection have a risk of bleeding complications ranging from 1.3% to 5.9%, which is much higher than the risk of nonfatal bleeding of 0.16% in nonuremic patients [26,27,28,29]. In addition, the biopsy specimens are prone to sampling and interpretation variability [30]. The use of noninvasive means to assess hepatic fibrosis in hemodialysis patients with HCV infection is appealing to healthcare providers, particularly in monitoring disease evolution over time. In this review, we will summarize the clinical performance of noninvasive indices to predict the stage of hepatic fibrosis in hemodialysis patients with HCV infection, and propose a pragmatic recommendation regarding the care for this special population based on current evidence.

## 2. Biochemical Index

### 2.1. Aspartate Transaminase (AST) to Alanine Transaminase (ALT) Ratio (AAR)

An elevated AAR has been known to suggest cirrhosis in nonuremic HCV patients, with a positive predictive value (PPV) and specificity of 100% when the cut-off value is ≥1 [31]. Ustündag et al. assessed the AAR features in 49 hemodialysis patients with HCV infection who underwent liver biopsy. They found that the AAR increased with more severe hepatic fibrosis (0.36 ± 0.17, 0.67 ± 0.17, and 0.86 ± 0.07 in patients with no fibrosis, mild fibrosis, and moderate fibrosis) [32]. Although the AAR can be of value in predicting the severity of hepatic fibrosis in hemodialysis patients with HCV infection, no patients exhibited cirrhosis in this study, making the AAR cut-off value of ≥1 to diagnose cirrhosis elusive in this special population.

Schmoyer et al. assessed the diagnostic power of the AAR in predicting significant hepatic fibrosis (≥F2), according to the METAVIR scores in hemodialysis patients with HCV infection, which revealed that the area under the receiver operating characteristic (AUROC) was only 0.59. The PPV and negative predictive value (NPV) were 27.0% and 92.3% at a cut-off value of 0.70 [33]. Because the AAR is designed to predict cirrhosis, which is seldom seen in hemodialysis patients, applying the AAR in predicting ≥F2 is of limited clinical utility (Table 1).

### 2.2. AST-to-Platelet Ratio Index (APRI)

Wai et al. correlated various biochemical parameters with the stage of hepatic fibrosis in 270 nonuremic patients with HCV infection. They found that the levels of platelet count, AST, ALT, and alkaline phosphatase (ALP) were highly associated with patients with ≥F2 and cirrhosis (F4). A novel biochemical index, APRI, was developed by amplifying the different effects of the platelet count and AST level on hepatic fibrosis stage [39]. The AUROCs were 0.88 and 0.94 in predicting HCV patients with a fibrosis stage of ≥F2 and F4, respectively. The sensitivity, specificity, PPV, and NPV for ≥F2 were 91%. 47%, 61%, and 86% with a cut-off value of 0.5, and 41%, 95%, 88%, and 64% with a cut-off value of 1.5. In addition, the sensitivity, specificity, PPV, and NPV for F4 were 89%, 75%, 38%, and 98% with a cut-off value of 1.0, and 57%, 93%, 57%, and 98% with a cut-off value of 2.0. Using these cut-off values, the clinicians can correctly diagnose 51% and 81% of patients with a fibrosis stage of ≥F2 and F4 by the APRI without requiring liver biopsy. The APRI has been widely applied in clinical practice because it is simple, readily available, and validated in meta-analyses [40].

Schiavon and Liu et al. independently assessed the diagnostic accuracy of the APRI in 203 and 209 hemodialysis patients with HCV infection, who received percutaneous liver biopsy [34,35]. The AUROCs to predict a fibrosis stage of ≥F2 were 0.80 and 0.83. The PPVs were 37% and 49%, and the NPVs were 93% and 85% at a cut-off value of 0.40. The PPVs were 66% and 82%, and the NPVs were 84% and 71% at a cut-off value of 0.95. The AUROC was 0.84 to predict a fibrosis stage of ≥F3 [34]. When the cut-off values were 0.40 and 0.95, the PPVs were 28% and 46%, and the NPVs were 87% and 83% [36]. If the cut-off values were 0.55 and 1.00, the PPVs were 24% and 29%, and the NPVs were 99% and 94% [34] (Table 1).

Schmoyer et al. validated the performance of the APRI in 139 hemodialysis patients with HCV infection and found that the AUROC to predict a fibrosis stage of ≥F2 was 0.68, which was lower than Schiavon’s and Liu’s reports. The AUROC in patients with elevated ALT levels was higher than that in patients with normal ALT levels (0.74 versus 0.42), if the clinicians defined the normal limits of ALT as 35 U/L for men and 25 U/L for women [33]. This finding followed Liu’s finding that the diagnostic accuracy of the APRI at off-therapy follow-up tended to decrease in patients who achieved sustained virologic response (SVR) compared to those who did not, probably due to the rapid normalization of AST and ALT levels in SVR patients [35,41] (Table 1).

Based on the APRI results, the fibrosis stage can be correctly diagnosed without requiring liver biopsy in around 50% of hemodialysis patients with HCV infection [34,35,36]. Because the concentration of pyridoxin-5’-phosphate, a cofactor required in the full catalytic activity of AST and ALT, is significantly reduced in hemodialysis patients, the APRI cut-off values to stage hepatic fibrosis are lower in hemodialysis patients than in nonuremic patients [42].

Pestana and Lee et al. independently evaluated the clinical utility of APRI in 70 and 116 hemodialysis patients with HCV infection, taking transient elastography (TE) as the reference standard. They used the same cut-off values of liver stiffness as those in nonuremic patients to stage hepatic fibrosis [37,38] (Table 1). The AUROCs of the APRI in predicting patients with a fibrosis stage of ≥F2, ≥F3, and F4 ranged from 0.70 to 0.80, which were lower than the AUROCs in studies that used liver biopsy as the reference standard. The selected cut-off values of the APRI were lower than in Schiavon’s and Liu’s reports, implying that the correlation between the APRI and TE was inferior to that between the APRI and liver histology. The clinicians were unable to identify the severity of hepatic fibrosis in hemodialysis patients with HCV infection if they determined the fibrosis stage by the APRI with the cut-off values for nonuremic patients (0.50 and 1.50 for ≥F2; 1.00 and 2.00 for F4) [38,39].

### 2.3. Fibrosis Index Based on Four Parameters (FIB-4)

FIB-4, an index that combined four biochemical parameters including age, AST, ALT, and platelet count, was initially developed to predict the severity of hepatic fibrosis in 832 patients with HCV and human immunodeficiency virus (HIV) coinfection [43]. The AUROC in predicting a fibrosis stage of ≥F3 was 0.765. The NPV and PPV were 90% and 65% at cut-off values of <1.45 and >3.25, and 87% of patients with a FIB-4 score outside 1.45 to 3.25 can avoid fibrosis staging with liver biopsy. Subsequently, Vallet-Pichard et al. validated the performance of FIB-4 in 847 patients with HCV monoinfection, which yielded an AUROC of 0.85 in predicting a fibrosis stage of ≥F3. The NPV and PPV at cut-off values of <1.45 and >3.25 were 94.7% and 82.1%, and 72.8% patients with a fibrosis stage of <F3 or ≥F3 can be correctly classified with FIB-4 [44].

Schmoyer et al. showed that the AUROC of FIB-4 to predict hemodialysis patients with HCV infection with a fibrosis stage of ≥F2 was 0.71, which was comparable to the AUROC of the APRI (0.68) [33]. The PPV and NPV were 43.9% and 83.7% when the FIB-4 cut-off value was 2.13. Using TE as the reference standard, Pestana and Lee et al. evaluated the role of FIB-4 in diagnosing different stages of hepatic fibrosis, which revealed that the AUROCs were 0.79 and 0.68 in predicting a fibrosis stage of ≥F2, and 0.85 and 0.77 in predicting a fibrosis stage of F4, respectively [37,38]. However, wide AUROC and cut-off value variations existed, making the clinical utility of FIB-4 to diagnose the severity of hepatic fibrosis in hemodialysis patients with HCV infection elusive (Table 1). Because the FIB-4 index in hemodialysis patients with HCV infection would be expected to be lower than in nonuremic HCV patients, as is observed in the APRI, it is not practical to apply the conventional cut-off values of 1.45 and 3.25 in hemodialysis patients with HCV infection [33,37,38].

### 2.4. King’s Score and Fibrosis Index

In addition to the AAR, APRI, and FIB-4 indices, Schmoyer et al. applied King’s score and the Fibrosis index, which have been tested in nonuremic patients with HCV, to diagnose the severity of hepatic fibrosis in hemodialysis patients with HCV infection [45,46]. The AUROC of King’s score in predicting a fibrosis stage of ≥F2 was 0.69, which was inferior to the AUROC of 0.79 to predict a similar stage of hepatic fibrosis in nonuremic patients. The cut-off value of a King’s score of 6.9 had an NPV of 87.3% for a fibrosis stage of F2, compared to that of 12.3 with an NPV of 77% in nonuremic patients [33,45]. Similarly, the AUROC of the Fibrosis index in predicting a fibrosis stage of ≥F2 was 0.59, and was also lower than the AUROC of 0.85 in nonuremic patients. There was a great disparity of the selective cut-off values for the Fibrosis index, with 10.39 in hemodialysis patients and 2.1 in nonuremic patients to reach NPVs of 84.7% and 78.8%, respectively [33,46] (Table 1).

## 3. Serological Index

### 3.1. FibroTest

In early 2000, the MULTIVIRC group developed a novel index, named FibroTest, to grade the severity of hepatic fibrosis in patients with HCV infection. They combined α2 macroglobulin, haptoglobin, apolipoprotein A1, γ-glutamyl transpeptidase, total bilirubin, age, and sex into a regression model to reach a final score ranging from 0.00 to 1.00 [47]. The same group further confirmed that only 87 of 537 (16.2%) patients included in another prospective study had discordant results for fibrosis stage between FibroTest and liver biopsy. Furthermore, kidney failure did not significantly contribute to inconsistent fibrosis results [48]. Although the diagnostic accuracy of FibroTest showed promise to assess hepatic fibrosis in HCV, a small-scaled study conducted by the same group that enrolled 50 hemodialysis patients with HCV infection, showed that the AUROCs of FibroTest were only 0.47 and 0.66 in predicting patients with a fibrosis stage of ≥F2 and ≥F3, respectively [49]. An independent study that recruited 33 hemodialysis patients with HCV infection also showed an AUROC of only 0.45 in predicting a fibrosis stage of ≥F2 [50] (Table 2).

### 3.2. Hyaluronic Acid (HA)

HA is a chief extracellular matrix (ECM) component and continues to deposit in the liver in response to hepatic inflammation, leading to hepatic fibrosis or cirrhosis [55]. Serum levels of HA correlate with the severity of hepatic fibrosis in nonuremic patients with HCV infection, particularly in those with advanced liver diseases [56,57,58,59]. A cut-off value of 60 ng/mL had NPVs of 93% and 99% for patients with ≥F3 and F4, respectively, while a cut-off value of 72 ng/mL had a PVV of 100% for those with F4 [58,59].

Schiavon et al. assessed the utility of HA in 185 hemodialysis patients with HCV infection, which revealed a modest AUROC of 0.65 in predicting a fibrosis stage of ≥F2. Although the serum HA level of 64 ng/mL had an NPV of 86%, the PPV was only 42% at a cut-off value of 205 mg/mL [51]. Avila et al. conducted a small-scaled study that recruited 23 hemodialysis patients with HCV infection. In contrast to Schiavon’s finding, the AUROC was 0.81 in predicting a fibrosis stage of ≥F2. However, the PPV was only 79%, even though they set the cut-off value of HA to 984.8 ng/mL [52]. The significant discrepancy between both studies may be attributed to the limited number and the heterogeneity of the patients who also had hepatitis B virus (HBV) coinfection, drug-induced liver injury (DILI), or autoimmune hepatitis (AIH) in Avila’s study (Table 2). Orăşan et al. reported the diagnostic value of HA in 38 hemodialysis patients with HCV infection, taking TE as the reference standard. Although the NPV was 80% when the cut-off value of HA was 39.72 ng/mL for a fibrosis stage of ≥F2, the PPV was only 80% when the cut-off value of HA was 88.56 ng/mL for a fibrosis stage of ≥F3 or F4 [53].

Since HA is not specific to liver fibrosis, the serum levels of HA in hemodialysis patients are expected to be higher that nonuremic patients because of the coexistence of systemic inflammatory/fibrotic reactions. Based on the published data, HA is of little clinical utility in predicting the severity of hepatic fibrosis in hemodialysis patients with HCV infection.

### 3.3. Tyrosine-Lysine-Leucine 40 Kilodalton (YKL-40)

YKL-40, also known as chitinase-3-like protein 1 (CHI3L1), is a glycoprotein expressed and secreted by various cells, including macrophages, chondrocytes, fibroblast-like synovial cells, hepatic stellate cells, and vascular smooth muscle cells. The serum levels of YKL-40 correlate with the severity of hepatic fibrosis of various etiologies [60]. In nonuremic patients with HCV infection, Saitou et al. demonstrated that the AUROCs of YKL-40 were 0.809 and 0.795 in predicting a fibrotic stage of ≥F2 and F4 [61]. The PPV and NPV were 80% and 79% in predicting patients with ≥F2 hepatic fibrosis when the cut-off level of YKL-40 was 186.4 ng/mL, and were 73% and 78% in predicting patients with cirrhosis when the cut-off level was 284.8 ng/mL.

In contrast to the high AUROC of YKL-40 in assessing the stage of hepatic fibrosis in HCV patients without kidney failures, Schiavon et al. showed that the AUROC of YKL-40 in predicting a fibrosis stage of ≥F2 in hemodialysis patients with HCV infection was only 0.607 [51]. Despite the NPV being 84% at a cut-off value of 290 ng/mL, the PPV remained only 35% at a cut-off value of 520 ng/mL (Table 2). Furthermore, Tatar et al. also confirmed a poor correlation of YKL-40 with hepatic fibrosis in these patients [54]. As with HA, systemic tissue inflammation/fibrosis other than that originating from HCV infection is commonly seen in hemodialysis patients, making YKL-40 of limited usefulness in diagnosing hepatic fibrosis [62].

## 4. Radiological Index

### Transient Elastography (TE, FibroScan)

TE is a noninvasive tool that assesses hepatic fibrosis by measuring liver stiffness [63]. To date, TE has been extensively validated in nonuremic patients with HCV infection, with a diagnostic power at least equivalent to various biochemical and serological indices. The AUROCs in predicting a fibrosis stage of ≥F2, ≥F3, and F4 are 0.83, 0.90, and 0.95, respectively [64]. The PPV and NPV are 95% and 48% at a cut-off value of 7.1 kilopascal (kPa), 87% and 81% at a cut-off value of 9.5 kPa, and 77% and 95% at a cut-off value of 12.5 kPa to predict patients with ≥F2, ≥F3, and F4. About 5% of patients, of whom most are obese, may fail to reach reliable results. Appling the XL probe may improve the diagnostic yield of TE in obese patients.

Liu et al. prospectively assessed hepatic fibrosis with TE in 284 hemodialysis patients with HCV infection who received liver biopsy. The AUROCs of TE in predicting a fibrosis stage of ≥F2, ≥F3, and F4 were 0.96, 0.98, and 0.99, which were significantly higher than the AUROCs of the APRI [65]. The PPVs reached 89%, 95%, and 100% when the cut-off values were 7.1 kPa, 9.5 kPa, and 12.5 kPa, and approximately 90% of patients did not require liver biopsy (Table 3). A large-scaled study confirmed the excellent performance of TE, which showed a very similar distribution of fibrosis stage in 659 hemodialysis patients, based on the liver stiffness measurement with TE [66]. Although no studies compare the diagnostic accuracy of TE between hemodialysis and nonuremic patients with HCV infection, the AUROCs of TE tend to increase by 0.05 in hemodialysis patients with HCV infection, compared to those in nonuremic patients [64,65]. Prior studies have shown that increased ALT levels, a surrogate marker of liver inflammation, may overestimate liver stiffness by increasing the spleno–portal flow [67,68,69]. The lower ALT levels in hemodialysis patients with HCV infection than those in nonuremic patients may contribute to the better performance of TE in diagnosing the severity of hepatic fibrosis [70].

Because the portal flow increases in hemodialysis patients with food intake and excess fluid accumulation, clinicians should perform TE in hemodialysis patients who are fasting and complete a session of hemodialysis to avoid overestimating the severity of hepatic fibrosis [65,71,72,73,74].

## 5. Clinical Application of Noninvasive Indices for Hepatic Fibrosis in Hemodialysis Patients with HCV Infection

A pragmatic recommendation of applying noninvasive indices based on the diagnostic accuracy in predicting the severity of hepatitis fibrosis is depicted, to optimize the clinical practice for hemodialysis patients with HCV infection (Table 4).

In medical institutions where TE is readily available and accessible, directly measuring liver stiffness by this sonography-based technique can offer an excellent diagnostic yield, to predict the severity of hepatic fibrosis and avoid invasive liver biopsy in up to 90% of hemodialysis patients with HCV infection [65]. Because liver stiffness measurement is not a synonym of liver fibrosis, patient-related confounding factors that may alter the liver stiffness, such as heart failure-induced hepatic congestion, hepatic necroinflammatory reaction, digestion state, etc., should be taken into consideration with TE [75]. Therefore, patients are recommended to receive TE at fasting state and after hemodialysis. Magnetic resonance (MR) elastography, an advanced technique that can comprehensively assess the stiffness of the whole liver, has been shown to yield higher diagnostic accuracy than TE in nonuremic patients [76]. Although TE has been shown to perform better than biochemical or serological indices in hemodialysis patients with HCV infection, studies aiming at the clinical performance of MR elastography are awaited in this special population.

For medical institutions where TE is unavailable, the APRI can be the choice to stage hepatic fibrosis because of its ease of use and access. Three independent studies, which adopted liver biopsy as the reference standard, have shown that the AUROCs of the APRI were 0.80 to 0.84 in predicting a fibrosis stage of ≥F2 and ≥F3. An APRI of 0.40 and 0.95 can have NPVs of 85% to 93% and PPVs of 66% to 82% to predict F2 [34,35,36]. Because the percentage of hemodialysis patients with a fibrosis stage of ≥F3 is limited, the clinical value of the APRI would be more focused on the high NPV for F3. Current data indicate that an APRI of <0.55 can exclude a fibrosis stage of ≥F3 in 99% of hemodialysis patients with HCV infection [34].

Although the FIB-4 index has superior diagnostic accuracy to the APRI to predict the severity of hepatic fibrosis in nonuremic HCV patients, the performance of FIB-4 in hemodialysis patients with HCV infection is not ideal or stable [33,37,42]. Furthermore, we do not recommend King’s score or the Fibrosis index in these patients, based on the poor diagnostic performance [33].

Because most hemodialysis patients present with systemic inflammation/fibrosis from nonhepatic origins, all serological indices targeting ECM dynamics, including FibroTest, hyaluronic acid, and YKL-40, are not recommended in clinical practice to predict the stage of hepatic fibrosis in hemodialysis patients with HCV infection.

To date, data regarding the application to monitor the evolution of fibrotic changes following antiviral treatment are scarce. In nonuremic patients with HCV who achieve SVR with antiviral therapy, studies have shown that the APRI, FIB-4, and TE had low diagnostic accuracies in assessing the evolution of hepatic fibrosis [77,78]. Current evidence does not favor the use of the APRI to monitor the evolution of hepatic fibrosis because the diagnostic accuracy of the APRI in hemodialysis patients with HCV infection seemed to decrease once SVR was achieved with antiviral therapy, compared to the pretreatment status [35]. The clinical performance of TE or MR elastography to follow the evolution of hepatic fibrosis needs further investigation.

## 6. Conclusions

HCV infection remains prevalent in patients receiving hemodialysis. The introduction of DAAs has revolutionized the care of HCV in this special clinical setting, based on the excellent viral clearance rate and tolerance. Assessing hepatic fibrosis before and after antiviral treatment is essential for therapeutic and prognostic implications. Although percutaneous liver biopsy is the gold standard for assessing liver histology in patients with chronic liver diseases, the postprocedural complications, as well as the sampling and interpretation variability, limit the widespread use of this invasive technique. Because a noninvasive index of hepatic fibrosis has a preponderance of safety and a potential to monitor disease evolution by repeated measurements, it would be of great help in diagnosing the stage of hepatic fibrosis in hemodialysis patients with HCV infection.

Current evidence suggests that TE is the preferred tool to stage hepatic fibrosis in hemodialysis patients with HCV infection. Although the diagnostic accuracy of TE is higher than other noninvasive indices, only one study has been published to date. Therefore, independent research to validate the performance of TE is still needed. The APRI may be feasible in these patients when TE is not available or accessible. We do not recommend the AAR, FIB-4, King’s score, Fibrosis index, FibroTest, hyaluronic acid, and YKL-40 in assessing the severity of hepatic fibrosis in these patients because of low diagnostic yields. However, the roles of King’s score and the Fibrosis index remain uncertain because the number of studies is limited. More work is needed to confirm the feasibility of applying noninvasive indices to monitor hepatic fibrosis evolution in hemodialysis patients with HCV infection.

## Figures and Tables

**Table 1:** Summary of biochemical indices to predict hepatic fibrosis in hemodialysis patients with HCV infection.

Index ^a^	Authors, Year	Patient Number	Reference Standard	Fibrosis Distribution, n (%)	Main Findings	Limitation
AAR	Ustündag et al., 2000 [32]	49	liver biopsy	F0: 36 (73.5) F1: 8 (16.3) F2–F3: 5 (10.2)	F0: 0.36 ± 0.17; F1: 0.67 ± 0.17; F2-F3:0.86 ± 0.07 An increasing AAR with an increasing stage of hepatic fibrosis	No cirrhosis was identified.
	Schmoyer et al., 2020 [33]	139	liver biopsy	F0–F1: 104 (75.5) F2–F4: 35 (24.5)	AUROC of ≥F2: 0.59 (95% CI: 0.48–0.69) Cut-off value of 0.70 for ≥F2 PPV 27.0%; NPV 92.3%	-
APRI	Schiavon et al., 2007 [34]	203	liver biopsy	F0: 73 (36.0) F1: 82 (40.3) F2: 29 (14.3) F3: 13 (6.4) F4: 6 (3.0)	AUROC ≥F2: 0.801 ± 0.038 ≥F3: 0.844 ± 0.034 Cut-off value of 0.40 for ≥F2 PPV 37%; NPV 93% Cut-off value of 0.95 for ≥F2 PPV 66%; NPV 84% Cut-off value of 0.55 for ≥F3 PPV 24%; NPV 99% Cut-off value of 1.00 for ≥F3 PPV 29%; NPV 94%	The prediction model for ≥F3 was less accurate because of low index cases.
	Liu et al., 2010 [35]	279	liver biopsy	F0: 82 (29.4) F1: 96 (34.4) F2: 64 (22.9) F3: 25 (9.0) F4: 12 (4.3)	AUROC of ≥F2 Baseline: 0.83 (95% CI: 0.79–0.88) Follow-up: 0.71 (95% CI: 0.65–0.77) Follow-up (SVR): 0.75 (95% CI: 0.69–0.81) Follow-up (non-SVR): 0.80 (95% CI: 0.69–0.91) Cut-off value for ≥F2 (baseline) 0.40: PPV 49%; NPV 85% 0.80: PPV 83%; NPV 75% 0.95: PPV 82%; NPV 71% Cut-off value for ≥F2 (with SVR) 0.30: PPV 76%; NPV 81% 0.60: PPV 100%; NPV 70%	-
	Jiang et al., 2014 [36]	254 ^b^	liver biopsy	F0: 45 (18) F1: 93 (37) F2: 70 (27) F3: 36 (14) F4: 10 (4)	Cut-off value of 0.40 for ≥F3 PPV 28%; NPV 87% Cut-off value of 0.95 for ≥F3 PPV 46%; NPV 83%	The prediction model for ≥F3 was less accurate because of low index cases.
	Schmoyer et al., 2020 [33]	139	liver biopsy	F0–F1: 104 (75.5) F2–F4: 35 (24.5)	AUROC of ≥F2: 0.68 (95% CI: 0.59–0.79) Cut-off value of 0.49 for ≥F2 PPV 50.0%; NPV 84.5%	-
	Pestana et al., 2021 [37]	70	transient elastography	F0–F1: 35 (50) F2: 16 (23) F3: 5 (7) F4: 14 (20)	AUROC ≥F2: 0.73 (95% CI: 0.61–0.83) F4: 0.82 (95% CI: 0.71–0.90) Cut-off value for ≥F2 ≤0.25: NPV 81.8% >0.61: PPV 81.8% Cut-off value for F4 ≤0.42: NPV 97% >0.73: PPV 60%	Liver biopsy was not the reference standard.
	Lee et al., 2020 [38]	116	transient elastography	F0–F1: 46 (39.6) F2: 35 (30.2) F3: 11 (9.5) F4: 24 (20.7)	AUROC ≥F2: 0.70 (95% CI: 0.60–0.80) ≥F3: 0.73 (95% CI: 0.63–0.82) F4: 0.76 (95% CI: 0.66–0.86) Cut-off value of 0.24 for ≥F2 PPV 72.5%; NPV 68.6% Cut-off value of 0.25 for ≥F3 PPV 43.4%; NPV 94.9% Cut-off value of 0.28 for F4 PPV 33.3%; NPV 97.8%	Liver biopsy was not the reference standard.
FIB-4	Schmoyer et al., 2020 [33]	139	liver biopsy	F0–F1: 104 (75.5) F2–F4: 35 (24.5)	AUROC of ≥F2: 0.71 (95% CI: 0.61–0.80) Cut-off value of 2.13 for ≥F2 PPV 43.9%; NPV 83.7%	-
	Pestana et al., 2021 [37]	70	transient elastography	F0–F1: 35 (50) F2: 16 (23) F3: 5 (7) F4: 14 (20)	AUROC ≥F2: 0.79 (95% CI: 0.68–0.88) F4: 0.85 (95% CI: 0.75–0.93) Cut-off value for ≥F2 ≤0.60: NPV 90.9% 1.87: PPV 87.5% Cut-off value for F4 ≤1.40: NPV 97.5% >2.22: PPV 70%	Liver biopsy was not the reference standard.
	Lee et al., 2020 [38]	116	transient elastography	F0–F1: 46 (39.6) F2: 35 (30.2) F3: 11 (9.5) F4: 24 (20.7)	AUROC ≥F2: 0.68 (95% CI: 0.58–0.78) ≥F3: 0.75 (95% CI: 0.66–0.85) F4: 0.77 (95% CI: 0.67–0.88) Cut-off value of 1.89 for ≥F2 PPV 76.8%; NPV 55.9% Cut-off value of 1.89 for ≥F3 PPV 50.1%; NPV 88.1% Cut-off value of 1.91 for F4 PPV 37.0%; NPV 93.4%	Liver biopsy was not the reference standard.
King’s score	Schmoyer et al., 2020 [33]	139	liver biopsy	F0–F1: 104 (75.5) F2–F4: 35 (24.5)	AUROC of ≥F2: 0.69 (95% CI: 0.58–0.80) Cut-off value of 6.9 for ≥F2 PPV 13.5%; NPV 87.3%	-
Fibrosis index	Schmoyer et al., 2020 [33]	139	liver biopsy	F0–F1: 104 (75.5) F2–F4: 35 (24.5)	AUROC of ≥F2: 0.59 (95% CI: 0.46–0.72) Cut-off value of 10.39 for ≥F2 PPV 23.1%; NPV 84.7%	-

AAR, aspartate transaminase to alanine transaminase ratio; APRI, aspartate transaminase-to-platelet ratio index; FIB-4, fibrosis index based on four parameters; AUROC, area under the receiver operating characteristic; NPV, negative predictive value; PPV, positive predictive value; CI, confidence interval. ^a^ The formula of each index was calculated as follows: AAR = AST (U/L)/ALT (U/L); APRI = AST (times of upper limit of normal)/platelet count (10^9^/L) × 100; FIB-4 = Age (year) × AST (U/L)/platelet count (10^9^/L)/ALT (U/L)^1/2^; King’s score = Age (year) × AST (U/L) × INR (international normalized ratio)/platelet count (10^9^/L); Fibrosis index = 8 − 0.01 × platelet count (10^9^/L) – albumin (g/dL). ^b^ Two hundred and twenty patients had APRI data.

**Table 2:** Summary of serological indices to predict hepatic fibrosis in hemodialysis patients with HCV infection.

Index	Authors, Year	Patient Number	Reference Standard	Fibrosis Distribution, n (%)	Main Findings	Limitation
FibroTest ^a^	Varaut et al., 2005 [49]	50	liver biopsy	F0: 1 (2) F1: 28 (56) F2: 11 (22) F3: 7 (14) F4: 3 (6)	AUROC ≥F2: 0.47 ≥F3: 0.66 Cut-off value of 0.20 for ≥F2 - PPV 52%; NPV 71% Cut-off value of 0.60 for ≥F2 - PPV 75%; NPV 61% Cut-off value of 0.20 for ≥F3 - PPV 31%; NPV 90% Cut-off value of 0.60 for ≥F3 - PPV 75%; NPV 83%	The case number was limited.
	Canbakan et al., 2011 [50]	33	liver biopsy	F0–F1: 17 (51.5) F2–F4: 16 (48.5)	AUROC of ≥F2: 0.45 (95% CI: 0.25–0.67) Cut-off value of 0.20 for ≥F2 - NPV 45% Cut-off value of 0.60 for ≥F2 - PPV 20%	The case number was limited.
Hyaluronic acid	Schiavon et al., 2008 [51]	185	liver biopsy	F0–F1: 140 (76) F2–F4: 45 (24)	AUROC of ≥F2: 0.650 ± 0.049 Cut-off value of 64 ng/mL for ≥F2 PPV 30%; NPV 86% Cut-off value of 205 ng/mL for ≥F2 PPV 42%; NPV 80%	-
	Avila et al., 2010 [52]	23	liver biopsy	F0–F1: 10 (43.5) F2–F4: 13 (56.5)	AUROC of ≥F2: 0.808 (95% CI: 0.662–0.995) Cut-off value of 984.8 ng/mL for ≥F2 Sensitivity 83.0%; specificity 70.0% PPV 79%; NPV 78%	The case number was limited. Including heterogeneous patients, such as HBV coinfection, DILI, and AIH.
	Orăşan et al., 2015 [53]	38	transient elastography	F1: 20 (52.6) F2: 6 (15.8) F3: 4 (10.5) F4: 8 (21.1)	Cut-off value of 39.72 ng/mL for ≥F2 PPV 68%; NPV 80% Cut-off value of 88.56 for ≥F3 PPV 80%; NPV 82% Cut-off value of 88.56 for F4 PPV 80%; NPV 82%	The case number was limited. Liver biopsy was not the reference standard. No patients presented with F0.
YKL-40	Schiavon et al., 2008 [51]	185	liver biopsy	F0–F1: 140 (76) F2–F4: 45 (24)	AUROC of ≥F2: 0.607 ± 0.050 Cut-off value of 290 ng/mL for ≥F2 PPV 28%; NPV 84% Cut-off value of 520 ng/mL for ≥F2 PPV 35%; NPV 79%	-
	Tatar et al., 2017 [54]	15	liver biopsy	F0–F1: 10 (67) F2–F4: 5 (33)	YKL-40 was not independently associated with the severity of hepatic fibrosis	The case number was limited.

YKL-40, tyrosine-lysine-leucine 40 kilodalton; AUROC, area under the receiver operating characteristic; NPV, negative predictive value; PPV, positive predictive value; CI, confidence interval; HBV, hepatitis B virus; DILI, drug-induced liver injury; AIH, autoimmune hepatitis. ^a^ The formula was calculated as follows: FibroTest = 1/(1 + e^−z^), where the z score = 4.467 × log_10_ [α2 macroglobulin (g/L)] − 1.357 × log_10_ [haptoglobin (g/L)] + 1.017 × log_10_ [γ-glutamyl transpeptidase (IU/L)] + 0.0281 × [age] + 1.737 × log_10_ [total bilirubin (𝜇mol/L)] − 1.184 × log_10_ [apolipoprotein A1(g/L)] + 0.301 × [sex; male = 1 and female = 0].

**Table 3:** Summary of radiological index to predict hepatic fibrosis in hemodialysis patients with HCV infection.

Index	Authors, Year	Patient Number	Reference Standard	Fibrosis Distribution, n (%)	Main Findings	Limitation
Transient elastography (TE, FibroScan)	Liu et al., 2011 [65]	284	liver biopsy	F0: 81 (28.5) F1: 102 (35.9) F2: 61 (21.5) F3: 26 (9.2) F4: 14 (4.9)	AUROC ≥F2: 0.96 (95% CI: 0.94–0.98) ≥F3: 0.98 (95% CI: 0.97–1.00) F4: 0.99 (95% CI: 0.98–1.00) Cut-off value of 5.3 kPa for ≥F2 PPV 82%; NPV 96% Cut-off value of 7.1 kPa for ≥F2 PPV 89%; NPV 80% Cut-off value of 8.3 kPa for ≥F3 PPV 93%; NPV 99% Cut-off value of 9.5 kPa for ≥F3 PPV 95%; NPV 92% Cut-off value of 9.2 kPa for F4 PPV 58%; NPV 100% Cut-off value of 12.5 kPa for F4 PPV 100%; NPV 97%	-

AUROC, area under the receiver operating characteristic; NPV, negative predictive value; PPV, positive predictive value.

**Table 4:** Comparison of diagnostic accuracy, accessibility, and ease of use of various noninvasive indices to stage hepatic fibrosis in hemodialysis patients with HCV infection.

Order of Priority ^a^	Noninvasive Index	Diagnostic Accuracy ^b^	Accessibility	Ease of Use
1	Transient elastography (TE)	++++	+	+++
2	APRI	+++	+++	+++
3	FIB-4	++	+++	+++
	King’s score	++	+++	+++
	Hyaluronic acid (HA)	++	++	++
4	AAR	+	+++	+++
	Fibrosis index	+	+++	+++
	YKL-40	+	+	++
	FibroTest	+	+	+

AAR, aspartate transaminase to alanine transaminase ratio; APRI, aspartate transaminase-to-platelet ratio index; FIB-4, fibrosis index based on four parameters; YKL-40, tyrosine-lysine-leucine 40 kilodalton. ^a^ The order of priority was based on the rank of diagnostic accuracy in each noninvasive index. ^b^ The diagnostic accuracy was based on the AUROC level: ++++ (>0.90); +++ (0.80–0.90); ++ (0.70–0.80); + (<0.70).
